# The Relationship Between Physical Activity and Emotional Intelligence in College Students: The Mediating Role of Self-Efficacy

**DOI:** 10.3389/fpsyg.2020.00967

**Published:** 2020-06-09

**Authors:** Kun Wang, Ying Yang, Tingran Zhang, Yiyi Ouyang, Bin Liu, Jiong Luo

**Affiliations:** ^1^Research Centre for Activity Detoxification, College of Physical Education, Southwest University, Chongqing, China; ^2^College of Physical Education, Sichuan Agricultural University, Ya’an, China

**Keywords:** college students, physical activity, self-efficacy, emotional intelligence, mental health, mediation

## Abstract

**Background:**

College students are an inexhaustible driving force for social development, and college students with good physical and psychological qualities can better adapt to changes in the external environment. The purpose of this study was to explore the intrinsic relationship of physical activity and emotional intelligence among college students and to determine the primary role of self-efficacy in their relationships.

**Methods:**

Eight hundred thirty-five college students from two comprehensive universities in Southwest China, whose average age was 20.13 ± 1.06 years old, were investigated using the Physical Activity Rating Scale (PARS-3), Chinese Emotional Intelligence Scale (EIS), General Self-Efficacy Scale (GSES), and other measuring tools. SPSS 22.0 software was used to analyze and process the data with Independent sample *t*-test, One-way ANOVA, Pearson correlation analysis, and regression analysis, and Amos 21.0 software was used to build the structural equation model.

**Results:**

(1) In terms of physical activity amount, self-efficacy, and emotional intelligence, male college students scored higher than female students. Furthermore, college students in humanities and social sciences had lower self-efficacy. In contrast, senior students had the lowest levels of physical activity and self-efficacy, and there was no discipline or grade distribution difference in emotional intelligence. (2) Physical activity amount was positively correlated with emotional intelligence (*r* = 0.24, *P* < 0.001) and with self-efficacy (*r* = 0.26, *P* < 0.001), and self-efficacy was positively correlated with emotional intelligence in college students (*r* = 0.18, *P* < 0.001). (3) Self-efficacy played a partial mediating role between physical activity and emotional intelligence in college students (*ES* = 0.06).

**Conclusion:**

Regular physical activity can improve the self-efficacy and emotional intelligence of college students, and effectively promote the physical and mental development of students.

## Introduction

In the 1990s, the concept of emotional intelligence (EI) became a popular topic in psychology research. Salovey and Mayer (1990) and Mayer and Salovey (1997) proposed that emotional intelligence was the expression and evaluation of the emotions of oneself and others, the ability to regulate the emotions of oneself and others, and the ability to use emotions to solve practical problems. In other words, it was a comprehensive ability to perceive, evaluate, and express emotions accurately. At present, the understanding of emotional intelligence in academic circles at home and abroad mainly includes two aspects: emotional intelligence ability theory and emotional intelligence trait theory. The former views emotional intelligence as a kind of cognitive ability, which involves the cognition and processing of emotional information, and the latter views emotional intelligence as a personality trait associated with typical behavior (Bar-On and Parker, 2000; Petrides and Furnham, 2000; Lu et al., 2016). Studies showed that emotional intelligence has an important impact on individual well-being (Austin et al., 2005), academic achievement (O’Boyle et al., 2011), and physical health (Martins et al., 2010). Emotional intelligence is regarded as an essential factor affecting individual achievement levels, especially for college students (Liu and Jiao, 2017). College students are in a critical period of their lives, establishing a correct world outlook, outlook on life, and values. They are prone to feel pressure from many sources such as their learning, growth, and employment, so they are often prone to internal conflicts when dealing with emotions and interpersonal relationships (Feng and Zhan, 2015). In the long run, it is easy to cause some emotional disorders in individuals, which will have a negative impact mental health (Yuan and Zhang, 2007). In previous research on the emotional intelligence of college students, it was found that the development of emotional intelligence has an essential impact on the growth and development of college students (Liu and Jiao, 2017). College students with good emotional intelligence may better perceive the internal and external environment and guide their behaviors to improve their ability to cope with various challenges (Xiao and Hou, 2017).

### Self-Efficacy and Emotional Intelligence

Self-efficacy refers to an individual’s subjective assessment of his or her ability or mastery to complete a specific behavior in one particular situation, which can be used to explain the causes of motivation in certain cases and predict and explain the corresponding response, and it is also the psychological motivation behind an individual’s ability to sustain their self-regulation (Bandura, 1977, 1997). Cognitive-behavioral studies have shown that there is a close relationship between self-efficacy and emotional intelligence. Bandura (1997) believed that self-efficacy could influence the mind-body regulation system; if an individual believes they have an effect onself-processing their stress they thus exert an effect on their physical and mental health. Self-efficacy and positive emotions are considered to be two critical personal resources in dealing with stress (Chang et al., 2004); there is a significant correlation among self-efficacy, positive emotions, and effective coping styles (Papousek et al., 2010). It has been found in adolescents that there is a moderate positive correlation between self-efficacy and emotional management, that is, the stronger the self-efficacy of middle school students, the stronger their emotional management ability (Huo, 2008). Similarly, the higher the self-efficacy of college students, the better their performance in emotional intelligence tests (Liao, 2012). Thus, the hypotheses H1 was proposed: College students’ self-efficacy positively correlates with emotional intelligence.

### Physical Activity and Self-Efficacy

With the development of activity psychology, the positive benefits of physical activity for mental health have been widely recognized (Sexton et al., 1989; Xiao, 2015). Among these benefits, physical activity plays a crucial role in improving self-efficacy (Downs and Strachan, 2016). After vigorous running or cycling, an individual’s self-efficacy increases (McAuley et al., 1995). Research has found that the amount of physical activity has a significant effect on high school students’ academic mood and self-efficacy, and different physical activity intensities have significantly different effects on self-efficacy (Zhao et al., 2019). There is also a significant correlation between physical activity and self-efficacy in college students; the higher the degree of physical activity, the higher the self-efficacy (Hou and Yang, 2016). Therefore, the hypotheses H2 was proposed : Physical activity positively correlated with the self-efficacy of College students.

### Physical Activity and Emotional Intelligence

Individuals who exercise for a long time are less likely to develop anxiety problems such as general anxiety disorder, panic disorder, and social disorder. When physical activity reduces, they report an increased frequency of anxiety disorders (Si, 2008; Downs and Strachan, 2016). It can improve participants’ sports experience pleasure, provide social support, promote physiological changes, and enhance physical and mental comfort, thereby reducing anxiety and depression symptoms, improving life quality, and alleviating cognitive decline (Li et al., 2015). In their latest study, Ruiz-Ariza et al. (2019) found that cooperative high-intensity interval training has a significant impact on the creativity and emotional intelligence of middle-school students and can effectively improve the creativity, happiness, and social ability of inactive teenagers. Xu (1998) found that when young people actively participate in pleasant, non-competitive, or rhythmic physical activity, it will produce apparent short-term emotional effects, thereby promoting the formation of a good emotional state. Studies on adults found that physical activities significantly negatively correlates with negative emotional scores—the more adults engage in physical activity, the lower their negative emotional scores (Xu et al., 2003). A meta-analysis found that physical activity is closely related to emotional intelligence, and individual psychological characteristics are more marked after physical activity (Ubago-Jimenez et al., 2019). However, there are many subjective and objective factors involved in physical activity. If the psychological and emotional effects of physical activity cannot be differentiated and controlled, the results are often ambiguous. Therefore, researchers often add mediating variables to detect the mechanism of the effect of physical activity on mental health (Sheng et al., 2016). Studies showed that there were interactions among physical activity, self-efficacy, and mental health in middle school students, and that self-efficacy played a mediating role between physical activity and mental health (Sheng et al., 2016). Students with more physical activities have a stronger sense of self-efficacy and tend to have higher subjective well-being and interpersonal adaptability (Zhang et al., 2002). Therefore, the hypotheses H3 and H4 were proposed: Physical activity can promote emotional intelligence and there was a significant positive correlation between physical activity and emotional intelligence, and self-efficacy would play a mediating role in the relationship between physical activity and emotional intelligence.

In addition, whether there were gender differences in the emotional intelligence of college students was an important research topic in previous studies. Some studies have found significant differences in the emotional intelligence of male and female college students (Wan, 2012), while others have found no significant difference (Pan and Qian, 2012). Meanwhile, a survey of the current situation of college students’ emotional intelligence in Guizhou Province of China found that the overall emotional intelligence of boys was significantly higher than that of girls (Wan, 2012). However, other studies found that the total score of the emotional intelligence of girls was significantly higher than that of boys (Yu et al., 2012). It can be seen that there is still controversy about the gender difference in college students’ emotional intelligence. The inconsistent findings may have been influenced by the sample size, geographical differences, and ethnic diversity (Tang and Zhang, 2013).

In summary, there are correlations between physical activity, self-efficacy, and emotional intelligence. But at present, most of the research mainly focuses on the single relationship between physical activity and self-efficacy, self-efficacy, and emotional regulation (Zhao et al., 2017). Lack of a holistic discussion on the relationship between the three variables, which limits the generalizability and theoretical depth of the conclusion, and the specific role of self-efficacy between physical activity and emotional intelligence of college students is unknown. In view of this, this study selects students from two comprehensive universities in Southwest China as the research object, and takes self-efficacy as the guidance, to further explore the relationship between physical activity and emotional intelligence in college students, and further clarify the internal relationship between physical activity, self-efficacy, and emotional intelligence through a structural equation model, to promote the sound personality and physical and mental health of college students Coordinated development.

## Subjects and Methods

### Subjects

Using the method of random sampling, undergraduates came from two comprehensive universities in Southwest China and the samples involved the freshman to senior students in four grades. Inclusion and exclusion criteria for the sample selection was: (1) Full-time undergraduate students, (2) No physical disability or movement disorder, and (3) No mental illness or mental disorder. Based on previous studies, and in order to facilitate the establishment of an excellent structural equation model, a total of 1,000 questionnaires were issued and 948 collected, with a response rate of 94.80%; 113 invalid questionnaires were excluded, and 835 valid surveys remained, with an effective rate of 88.08%. Among them, there were 374 male students and 461 female students, from the Physical Education College (71), Art and Media College (154), Water Conservancy and Hydropower College (173), Food College (152), Life Science College (107), Electromechanical College (96), and Information Engineering College (82). The participants’ average age was 20.13 ± 1.06 years, height was 1.68 ± 0.09 m, and weight was 60.40 ± 12.15 kg (Table 1).

**TABLE 1:** Demographic characteristics of subjects (*n* = 835).

Variable		*M* ± *SD*/*N* (%)	Variable		*M* ± *SD*/*N* (%)
Gender	Male	374 (44.79%)	College	Sports	71 (8.50%)
	Female	461 (55.21%)		Electromechanics	96 (11.50%)
Age (years)		20.13 ± 1.06		Foods	152 (18.20%)
Height (m)		1.68 ± 0.09		Information Engineering	82 (9.82%)
Weight (kg)		60.40 ± 12.15		Life Science	107 (12.81%)
				Water Conservancy	173 (20.72%)
				Arts and Media	154 (18.44%)
			Grade	Freshman	303 (36.29%)
				Sophomore	338 (40.48%)
				Junior	119 (14.25%)
				Senior	75 (8.98%)

Questionnaire collection procedure: In order to ensure the quality and reliability of the questionnaire, after explaining the notes and requirements of the survey in detail to the students, the participants completed the questionnaire separately within 20 min according to their actual situation and recycled it on the spot. In addition, in order to facilitate the participants to fill in the questionnaire, the distribution and filling in of the questionnaire was carried out in the classroom during the break time. This research protocol was approved by the Ethics Committee of Southwest University Hospital, and written informed consent was obtained from all participants under the Declaration of Helsinki.

### Survey Protocol

#### Questionnaire Design and Reliability and Validity Test

Based on the large amount of research literature, a structured questionnaire design was organized in this study. In order to ensure that the questionnaire had a good test reliability, 50 students were randomly selected from a university to fill in the questionnaire before it was officially issued. After carefully explaining the notes and requirements for filling in the questionnaire, the students were required to adhere to the attitude of seeking truth from facts and fill in the corresponding number. Two weeks later, the same batch of students were asked to fill in the same questionnaire for the second time, and the results of the two fillings are compared and analyzed. We found that the results of the two fillers were consistent, and the retest coefficient was 0.84, indicating that the questionnaire had a high test reliability. The main contents of the questionnaire consisted of the following three components:

##### Physical activity rating scale (PARS-3)

The three-question test method revised by Liang (1994) was adopted, and the questions used focused on the physical activity intensity (e.g., “Do you think about the intensity of physical activity?”), activity time (e.g., “How long do you spend each time on physical activity?”), and frequency of physical activity (e.g., “How long do your activity every week?”). A 5-point Likert scale was used for quantification, with each item scored on a scale of 1−5. Physical activity score = activity intensity score × (activity time score - 1) × activity frequency score, score interval was from 0 to 100 points. According to the physical activity score, the physical activity level was divided into low, moderate, and high categories: low intensity ≤19, moderate-intensity 20–42, and high intensity ≥43. A preliminary test of the questionnaire showed that the test-retest reliability was high, and the correlation coefficient was *r* = 0.82 (Liang, 1994).

##### Chinese emotional intelligence scale (CEIS)

Based on the theory of Mayer and Salovey (1997) the Chinese EIS originally compiled by Schutte and revised by Wang and He (2002) was adopted. The scale contains a total of 33 questions, of which items 5, 28, and 33 are reverse-scored. There are four dimensions in total: emotional perception (11 items, such as “I can clearly understand the emotions that I experience”), self-emotion management (six items, such as “When I am in a good mood, it is easy for me to solve the problem”), emotional management of others (10 items, such as “I can discern other people’s emotions by observing facial expressions”), and emotional application (six items, such as “I can understand the non-verbal information that others have passed to me”). A 5-point Likert scale is used for quantification, with each item scored on a scale of 1−5 points according to the options of “strongly disagree,” “disagree,” “neither disagree nor agree,” “agree,” and “strongly agree.” The higher the score, the higher the emotional intelligence. In this research, according to the internal consistency test, Cronbach’s alpha coefficients were 0.89 for emotional perception, 0.86 for self-emotion management, 0.90 for emotional management of others, and 0.81 for emotional application. The overall Cronbach’s alpha coefficient of the scale was 0.89. Verification results for the measurement model were χ^2^/df = 1.83, RMSEA = 0.05, AGFI = 0.97, TLI = 0.98, CFI = 0.92, IFI = 0.98, and GFI = 0.93. This showed that the questionnaire had good validity and reliability.

##### General self-efficacy scale (GSES)

The GSES compiled by Schwarzer and modified by Wang et al. (2001) has the advantages of few questions and easy administration; it has been widely used in psychological assessment and psychological research on college and middle-school students. This scale is a unidimensional scale consisting of 10 questions, such as “If I try to do it, I can always solve the problem.” A 5-point Likert scale is used for quantification, with each item scored on a scale of 1−5 points according to the options of “strongly disagree,” “disagree,” “neither disagree nor agree,” “agree,” and “strongly agree.” The higher the score, the higher the self-efficacy. In this research, according to the internal consistency test, the Cronbach’s alpha coefficient of the scale was 0.83. Verification results for the measurement model were χ^2^/df = 2.18, RMSEA = 0.04, AGFI = 0.91, TLI = 0.98, CFI = 0.95, IFI = 0.97, and GFI = 0.94. This showed that the questionnaire had good validity and reliability (Sheng et al., 2016).

#### Statistical Analysis

This study used SSPS 21.0 software to analyze and process the data. Among them, the independent sample *t*-test was used to test the differences in physical activity, self-efficacy, and emotional intelligence of college students of different gender and disciplines. One-way analysis of variance was used to test the differences in physical activity, self-efficacy, and emotional intelligence among college students of different grades. At the same time, one-way analysis of variance was also used to test the differences in self-efficacy and emotional intelligence of college students with different amounts of physical activity; Pearson correlation analysis was used to test the correlation among college students’ physical activity, self-efficacy, and emotional intelligence. In addition, linear regression analysis was used to further examine the relationship among physical activity amount, self-efficacy, and emotional intelligence, and the mediating analysis of Baron and Kenny (1986) was used to explore the mediating role of self-efficacy between physical activity amount and emotional intelligence. Finally, the two-step procedural method proposed by Gerbing and Anderson (1988) and the structural equation model was used to test the mediating role of self-efficacy between physical activity amount and emotional intelligence of college students. The significance level of all indicators was set as α = 0.05.

## Results

### Common Method Bias Test

Harman’s single-factor test was used to investigate the common method of bias in this study. The measurement items of all variables were put together for an unrotated factor analysis; the results showed that there were eight factors whose eigenvalues were greater than one; and the variation explained by the first factor was 19.62%, less than the critical standard of 40%. It can be seen that the common method bias did not cause serious problems in this study.

### Demographic Differences in Physical Activity, Self-Efficacy, and Emotional Intelligence

The results of the *t-*test (Table 2) showed that in the comparison of gender, male college students had higher physical activity quantity (*t* = 2.44, *P* < 0.01), self-efficacy (*t* = 3.04, *P* < 0.01) and emotional intelligence (*t* = 2.45, *P* < 0.01) than female students. In the comparison of specialties, college students in natural science had better self-efficacy than those in social science (*t* = - 2.66, *P* < 0.01). At the same time, there was no significant difference in physical activity or emotional intelligence between natural and social science students (*P* > 0.05).

**TABLE 2:** Demographic differences in physical activity, self-efficacy, and emotional intelligence (*N* = 835).

Variable	Physical activity		Self-efficacy		Emotional intelligence	
			
	*M*	*SD*	*M*	*SD*	*M*	*SD*
**Male (a)**	27.94	20.26	27.24	11.10	64.75	14.28
**Female (b)**	24.86	15.18	24.94	10.70	62.41	13.33
*t*	2.44**		3.04**		2.45**	
Comparison	a > b		a > b		a > b	
**Natural (a)**	25.59	17.51	26.88	10.97	63.47	13.70
**Social (b)**	27.03	17.90	24.87	10.81	63.45	13.95
*t*	1.18		−2.66**		−0.02	
Comparison	–		a > b		–	
**Freshman (a)**	28.38	17.68	26.49	11.20	63.47	13.95
**Sophomore (b)**	26.06	17.70	25.40	10.67	63.69	13.66
**Junior (c)**	23.27	17.87	27.47	11.19	63.47	14.22
**Senior (d)**	17.32	11.51	21.40	9.03	59.68	12.27
*F*	4.79**		2.80*		0.66	
Multiple comparison	a > c; a > d; b > d		a > d; c > d		–	

*P < 0.05, **P < 0.01.

The results of ANOVA (Table 2) showed that in the comparison of grades, freshmen had significantly higher physical activity than juniors and seniors, and sophomores had higher activity than seniors (*F* = 4.79, *P* < 0.01); meanwhile, self-efficacy was significantly higher in the freshman and junior students than in the seniors (*F* = 2.80, *P* < 0.05). There were no significant differences in physical activity, self-efficacy, or emotional intelligence between the junior and senior students, no significant difference in physical activity among freshmen, sophomores, and juniors, and no significant difference in self-efficacy among the four grades (*P* > 0.05).

### Differences in Self-Efficacy and Emotional Intelligence Among College Students With Different Physical Activity

In order to further explore the differences in self-efficacy and emotional intelligence among college students with different amounts of physical exercise, the physical activity was divided into three levels according to previous studies (Liang, 1994) as follows: low activity amount (*n* = 335), moderate activity amount (*n* = 402), and high activity amount (*n* = 98).

The results of ANOVA (Table 3) showed that there was a significant difference in self-efficacy and emotional intelligence in college students with different physical activity levels. In terms of self-efficacy, the high-activity group was significantly better than the low-activity group and the moderate-activity group (*F* = 16.23, *P* < 0.001), while there was no significant difference between the low-activity group and the high-activity group (*P* > 0.05). In terms of emotional intelligence, the high-activity group was significantly better than the low-activity group and the moderate-activity group (*F* = 19.95, *P* < 0.001), while there was no significant difference between the low-activity group and the moderate-activity group (*P* > 0.05). The three dimensions of emotional perception (*F* = 32.89, *P* < 0.001), self-emotional management (*F* = 2.92, *P* < 0.05), and emotional management of others (*F* = 40.82, *P* < 0.001) all had the same results, but in terms of emotional application, there was no difference in the physical activity amount (*F* = 1.86, *P* > 0.05).

**TABLE 3:** Differences in self-efficacy and emotional intelligence among college students with different physical activity amount (*N* = 835).

Variable	Low PA (a)		Moderate PA (b)		High PA (c)		*F*	Multiple comparison
			
	*M*	*SD*	*M*	*SD*	*M*	*SD*		
Self-efficacy	26.13	11.16	24.52	8.81	31.40	15.46	16.23***	c > a, c > b
Emotional intelligence	61.53	12.49	63.16	12.98	71.28	18.15	19.95***	c > a, c > b
Emotional perception	14.79	3.38	15.26	3.82	18.82	8.13	32.89***	c > a, c > b
Self-emotional management	16.47	5.49	16.50	5.33	17.89	5.33	2.92*	c > a, c > b
Emotional management of others	12.44	2.39	12.86	2.58	15.85	6.91	40.82***	c > a, c > b
Emotional application	17.95	4.79	18.52	4.24	18.69	4.50	1.86	–

*P < 0.05, ***P < 0.001.

### Correlation and Regression Analysis of Physical Activity, Self-Efficacy, and Emotional Intelligence in College Students

The results of the correlation analysis (Table 4) showed that physical activity amount, self-efficacy, and emotional intelligence were significantly correlated among college students. Among them, the amount of physical activity and self-efficacy had a significant positive correlation (*r* = 0.26), physical activity amount and emotional intelligence had a significant positive correlation (*r* = 0.24), self-efficacy and emotional intelligence had a significant positive correlation (*r* = 0.18), and the correlation coefficients of the main variables were significant; thus, hypotheses H1, H2, and H3 of this study were effectively confirmed, and we thereby provided some theoretical basis for the subsequent tests of the mediating role of self-efficacy in the relationship of physical activity and emotional intelligence.

**TABLE 4:** Correlation and regression analysis of physical activity, self-efficacy, and emotional intelligence in college students.

	1	2	3	4	5	6	7	8
Gender (1)	–	0.09*	−0.11**	−0.09*	−0.10**	0.05	−0.07*	–0.03
Physical activity amount (2)		–	0.26***	0.24***	0.32***	0.10**	0.35***	0.03
Self-efficacy (3)			–	0.18***	0.25***	0.07	0.31***	–0.01
Emotional intelligence (4)				–	0.80***	0.80***	0.77***	0.70***
Emotional perception (5)					–	0.39***	0.71***	0.41***
Self-emotion management (6)						–	0.46***	0.51***
Emotional management of others (7)							–	0.34***
Emotional application (8)								–
*M*	1.55	26.24	25.97	63.46	15.49	16.65	13.04	18.31
*SD*	0.50	17.69	10.93	13.80	4.56	5.41	3.49	4.50

*P < 0.05, **P < 0.01, ***P < 0.001.

Linear regression analysis was used to test the effect of physical activity on emotional intelligence in college students, and the mediating effect test method of Baron and Kenny (1986) was adopted, which consists of three steps: First, independent variables have an impact on the dependent variable, and the regression coefficients reach a significance level. Second, independent variables affect mediating variables, and the regression coefficients are significant. Third, the combined effects of mediating variables and independent variables on the dependent variable reach a significance level. The influence of the mediating variable on the dependent variable must reach a significant level; at this time, if the influence of the independent variable on the dependent variable is significant, the mediating variable plays a complete mediating role. If the influence of the independent variable on the dependent variable is reduced, the mediating variable plays a partial mediating role.

The results of the regression analysis (Table 5) were as follows:

**TABLE 5:** Multivariate regression analysis of physical activity amount on emotional intelligence in college students (*N* = 835).

	Independent	Dependent	Beta	*t*	*P*	*R*	*R*^2^	*F*	*P*	*95% CI*
Equation 1	Physical activity amount	Emotional intelligence	0.24	7.25	0.000	0.24	0.06	52.53	0.000	(0.12, 0.26)
Equation 2	Physical activity amount	Self-efficacy	0.26	7.83	0.000	0.26	0.07	60.26	0.000	(0.10, 0.22)
Equation 3	Self-efficacy	Emotional intelligence	0.12	3.52	0.000	0.27	0.07	32.79	0.000	(0.04, 0.25)
	Physical activity amount		0.21	6.12	0.000					(0.10, 0.23)

Equation 1: When physical activity amount was used as the predictive variable and emotional intelligence as the dependent variable, the physical activity amount had a significant positive predictive power on emotional intelligence (β = 0.24, *F* = 52.53, *P* < 0.001).
Equation 2: When the physical activity amount was the predictive variable and self-efficacy was the dependent variable, the amount of physical activity had a significant positive predictive power on self-efficacy (β = 0.26, *F* = 60.26, *P* < 0.001).
Equation 3: When self-efficacy and physical activity amount acted together as predictive variables and emotional intelligence as the dependent variable, it can significantly predict emotional intelligence. Among them, self-efficacy had a significant positive predictive power on emotional intelligence (β = 0.12, *t* = 3.52, *P* < 0.001); the physical activity amount also had a significant positive predictive power on emotional intelligence (β = 0.21, *t* = 6.12, *P* < 0.001), but the regression coefficient of Equation 1 was reduced from 0.24 to 0.21, though it was still significant, indicating that self-efficacy played a partial mediating role in the relationship between physical activity amount and emotional intelligence. Thus, the hypotheses H4 of this study was conclusively confirmed.

### Test of Mediating Role of Self-Efficacy Between Physical Activity and Emotional Intelligence in College Students

**FIGURE 1:**
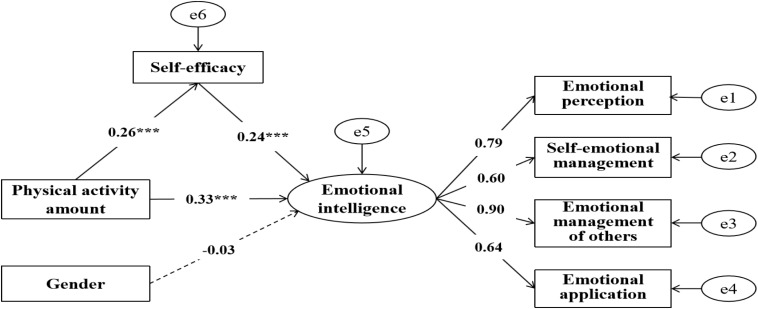
Model chart of the mediating power of self-efficacy on the relationship between physical activity amount and emotional intelligence.

The structured equation model was used to test the mediating power of self-efficacy on the relationship between physical activity and emotional intelligence (Figure 1). The fit indexes of the structured equation model were as follows: χ^2^/df = 16.70, RMSEA = 0.13, GFI = 0.93, IFI = 0.85, CFI = 0.85, and AGFI = 0.86. Owing to the larger sample size, a larger chi-square value appeared. Therefore, according to previous research viewpoints, the structural equation model was modified by modification indices. After the modification that the chi-square value of the model significantly reduced, all indicators of the model reached the ideal level, as follows: χ^2^/df = 1.48, RMSEA = 0.02, GFI = 0.99, IFI = 0.99, CFI = 0.99, and AGFI = 0.98, which indicated that the mediating test was suitable.

The structured equation model worked as follows: First, when emotional intelligence was the dependent variable and physical activity amount was the predictive variable, the path coefficient of the direct power of physical activity on emotional intelligence was significant (β1 = 0.37, *SE* = 0.01, *P* < 0.001). When the mediating variable of self-efficacy was added to the path of physical activity to emotional intelligence, the path coefficients of the relationship between physical activity and self-efficacy (β = 0.26, *SE* = 0.02, *P* < 0.001), the relationship between self-efficacy and emotional intelligence (β = 0.24, *SE* = 0.01, *P* < 0.001), and the relationship between physical activity amount and emotional intelligence (β2 = 0.33, *SE* = 0.01, *P* < 0.001) were significant. Notably, when we added the mediating variable, the path coefficient of the relationship between physical activity amount and emotional intelligence declined from 0.37 to 0.33. However, the path coefficient was still significant, which indicated that self-efficacy plays a partial mediating role in the relationship between physical activity and emotional intelligence, and the mediating force was 0.06. In order to explore the role of the gender variable in the model, we set it as a control variable in the model. The results showed that the path coefficient of the relationship between gender and emotional intelligence was not significant (β = −0.03, *SE* = 0.25, *P* > 0.05), and the path coefficient of the model was almost unchanged, which indicated that gender cannot serve as an effective control variable in this model—the associations of physical activity amount, self-efficacy, and emotional intelligence showed no gender differences (Table 6).

**TABLE 6:** Path weight coefficient statistics table (*N* = 835).

Hypothesis	Variable relationship	Boot SE	CR	Direct power	Mediation power
H1	PA→EI	0.01	8.28	0.33	
H2	PA→SE	0.02	7.81	0.26	
H3	SE→EI	0.01	6.62	0.24	
H4	PA→SE→EI				0.06

*PA, physical activity amount; EI, emotional intelligence; SE, self-efficacy.*

## Discussion

### Analysis of Demographic Factors of College Students’ Physical Activity, Self-Efficacy and Emotional Intelligence

This study found that there were significant demographic differences in the three variables of physical activity, self-efficacy, and emotional intelligence among college students. From the perspective of gender, males not only engaged in physical activity more than females but also had significantly higher self-efficacy and emotional intelligence. This is consistent with previous studies (Santos et al., 2005; Raudsepp, 2006). Luciana et al. (2014) found that there is a significant difference in individual body image between college students of different genders—boys are more active in maintaining physical and sports behaviors and have a higher level of physical activity, while girls are more concerned with their shape than boys. Therefore, boys are more likely to take the time and initiative to engage in physical activity, while girls are more likely to engage in activity passively to lose weight or maintain body size. Meanwhile, many studies have shown that there are significant gender differences in emotional intelligence and self-efficacy between college students of different genders. Bar-On (2000) found that males have better adaptability and greater optimism in stress management than females, while females perform better in emotional awareness. Wan (2012) found that the overall emotional intelligence of boys was significantly higher than that of girls. In addition, Bandura et al. (2003) research on adolescents between the ages of 14 and 21 showed that boys’ self-efficacy in regulating negative emotions was higher than that of girls, while girls’ self-efficacy in regulating positive emotions was higher than that of boys. Research suggests that the reasons for gender differences may be related to sociocultural factors. In traditional Chinese culture, men are more associated with stability, responsibility, and strength, which requires men to rationally control their emotions; the social control standards for women’s emotions are relatively loose, and their various emotional expressions are more easily supported and understood. In addition, college students are in a critical period of growth and development. Most boys have a clear life plan and goals and look forward to realizing their value through their own efforts. Therefore, they tend to have stronger self-efficacy and positive emotional intelligence. In terms of specialty classification, there was no significant difference between natural science specialties and humanities and social sciences in physical activity or emotional intelligence, but the former was higher than the latter in self-efficacy, which may be due to natural science students having more practical experience and stronger rational thinking and practical ability. In terms of grade distribution, different grades of college students showed significant differences in physical activity and self-efficacy. First, the freshmen engaged in significantly more physical activity than the juniors and seniors, and the sophomores engaged in significantly more activity than the seniors; the reason may be that freshman and sophomore students have lower academic pressure and are therefore more willing to maintain physical fitness and sports behavior to enhance their physique and health (Guo et al., 2017). Second, the senior students’ self-efficacy was the lowest, which may be due to the pressure of employment, as well as more complicated personal and social relationships; the self-recognition from the subjective to the objective may cause these students to show lower self-efficacy, which may be a subjective factor in the plight of employment after graduation in today’s college students.

### Direct Relationship Between Physical Activity and Emotional Intelligence in College Students

The results of this study showed that physical activity and emotional intelligence in college students were significantly positively correlated. The physical activity amount can positively predict emotional intelligence—the more college students’ physical activity amount, the higher their emotional intelligence scores, which was consistent with many studies (Li et al., 2009; Ladino et al., 2016). It is widely believed that physical activity itself could improve positive emotions and reduce negative emotions. The solidarity, interpersonal communication, or emotional expression provided by the sports environment during physical activity were conducive to the development of emotional intelligence. Participation in physical activities can promote the reduction of tension, anger, and depression, and positive self-improvement of overweight adolescents (Gao et al., 2017). Besides, this study found that the higher the amount of college students’ physical activity, the more it can promote the development of emotional intelligence. We found that self-emotion management and emotional management of others were better in college students who engaged in a high amount of physical activity than in those with low or moderate activity. Previous studies have shown that physical activity positively correlates with emotional intelligence, and college students with high physical activity have relatively higher emotional intelligence (Liao, 2012; Downs and Strachan, 2016). Meanwhile, by studying the relationship between physical activity and mental health, Jiang and Zhu (1997) found that a moderate or high amount of physical activity had greater positive effects on mental health in college or middle-school students. These findings support the results of our study, indicating that regular physical activity is essential to improving the emotional intelligence of college students and promoting the sound development of their personality.

### Self-Efficacy as a Mediator Between Physical Activity and Emotional Intelligence in College Students

By the regression analysis and structured equation model, taking the self-efficacy as a mediating variable in the relationship between physical activity and emotional intelligence in college students, the results showed that the physical activity amount was significantly positively correlated with emotional intelligence and self-efficacy, and self-efficacy was significantly positively correlated with emotional intelligence; moreover, the partial mediating power of self-efficacy was significant—that is, physical activity and emotional intelligence have both direct and indirect relationships in college students, and self-efficacy plays a mediating role in indirect relationships, and this overall relationship has nothing to do with gender, which was in accordance with previous studies (Hou and Yang, 2016; Gao et al., 2017). Some similar studies (Li, 2014) found that physical activity not only has a direct role in promoting positive emotion for middle-school students but also has a significant influence on stress tolerance, mental health, and interpersonal relationships in adolescence (Zhang, 2016), and activity such as tai chi also has positive benefits on emotion regulation in the elderly and can positively adjust emotions (Su, 2017). In addition, Caprara et al. (2008) found that people with higher confidence in emotional regulation can regulate their emotions through effective emotional regulation strategies.

In a study on the mediating effect of self-efficacy, Sheng et al. (2016) found that self-efficacy plays a mediating role in the relationship between physical activity and mental health in middle-school students. Specifically, when measuring the direct effects of physical activity, individuals will also make subjective evaluations themselves, which include whether they can stick to activity with self-confidence and self-cognition, and such efficacy evaluation is considered to play an important mediating role in the relationship between physical activity and positive psychological effects. Therefore, improving students’ cognition of sports behavior has been one of the main goals of the school physical education curriculum (Wang and Ma, 2014). For teenagers, the higher their self-efficacy, the greater their confidence in their ability to engage in physical activity, and the easier it is to convert the existing effects of physical activity into internalized rules on the basis of their own judgment of the perception and evaluation of the corresponding behavior capacity (Dong et al., 2018), so as to stimulate satisfaction, happiness, and pleasure in the social intercourse of physical activity; on the contrary, it is easy to cause negative emotional experience, resulting in emotional intelligence decline. Bandura (1997) indicated that if people are sure that a particular behavior will produce positive effects, they will have stronger self-efficacy and then engage in corresponding behaviors. To sum up, whether for boys or girls, the pleasant experience brought on by regular physical activity can not only effectively improve the emotional intelligence of college students but also improve their emotional regulation and behavioral cognition of themselves and others. Moreover, it promotes the self-efficacy of college students. It has positive benefits in enhancing social communication, improving personality, and building self-confidence, thus indirectly improving the emotional intelligence of college students. Therefore, it was reasonable and scientific that self-efficacy has a mediating role between physical activity and the emotional intelligence of college students. It can seem that it is necessary to actively carry out useful physical education courses on the university campus and cultivate students’ enthusiasm for participating in physical activity, which is an essential measure to promote the development of students’ physical and mental health.

## Conclusion and Limitations

### Conclusion

(1) In terms of physical activity amount, self-efficacy, and emotional intelligence, male college students scored higher than female students. Furthermore, college students in humanities and social sciences had lower self-efficacy. In contrast, senior students had the lowest levels of physical activity and self-efficacy, and there was no discipline or grade distribution difference in emotional intelligence.
(2) The physical activity amount positively correlated with emotional intelligence and self-efficacy, and self-efficacy positively correlated with emotional intelligence in college students.
(3) Self-efficacy played a partial mediating role in the relationship between physical activity and emotional intelligence in college students.

### Limitations and Further Research Directions

This study used a holistic perspective to explore the associations of physical activity, self-efficacy, and emotional intelligence in college students. It reflected the importance of appropriate physical activity and promotion of self-efficacy in the healthy development and success of college students, which provides further enrichment to activity psychology theory.

In order to further explore the deeper mechanisms behind these relationships, the following limitations can be addressed in follow-up research:

(1) Owing to the use of a cross-sectional methodology, direct evidence for a causal relationship between variables cannot be obtained, and further research on cross-lag design and experimental intervention is needed in the future.

(2) This study focused on the mediating variable of self-efficacy in the relationship between physical activity amount and emotional intelligence; more mediating or moderating variables can be explored in the future.

## Data Availability Statement

All datasets generated for this study are included in the article/supplementary material.

## Ethics Statement

The studies involving human participants were reviewed and approved by the Southwest University Hospital, China. The patients/participants provided their written informed consent to participate in this study.

## Author Contributions

All authors designed this study and contributed to and approved the final manuscript. KW and JL carried out the protocol and questionnaire survey. TZ recruited the individuals with drug addicts. YY and YO undertook the statistical analysis and graphical representation of the data. BL and JL revised the draft.

## Conflict of Interest

The authors declare that the research was conducted in the absence of any commercial or financial relationships that could be construed as a potential conflict of interest.
